# Rapid thermoforming of polycarbonate cell culture accessories from 3D printed molds

**DOI:** 10.1371/journal.pone.0361065

**Published:** 2026-10-26

**Authors:** Ganesh Malayath, Fei Wang, Chao Zhou, Nathaniel Huebsch

**Affiliations:** 1 Department of Biomedical Engineering, McKelvey School of Engineering, Washington University in St. Louis, St. Louis, Missouri, United States of America; 2 Center for Cardiovascular Research, Washington University in St. Louis, St. Louis, Missouri, United States of America; Children’s Hospital of Orange County, UNITED STATES OF AMERICA

## Abstract

Standard cell-culture ware is mass-produced in fixed designs, whereas many tissue-engineering workflows require custom inserts, lids, chambers, or patterned substrates, especially during early-stage research and method development. Conventional fabrication methods for cell culture accessories involve injection molding and machining, which are too costly and time-consuming to implement for producing custom designs in small batches, and/or while testing the usefulness of a new design before mass production. Here, we propose polycarbonate (PC) thermoforming as a solution for creating customized transparent and autoclavable accessories. We demonstrate that optimized preheating of PC overcomes issues with bubbling during thermoforming. The use of high heat deflection temperature (HDT) resins allows these PC devices to be thermoformed off molds created by Digital Light Processing (DLP) 3D prints, enabling rapid prototyping of the PC. Using this approach, we fabricated custom PC well plate inserts, staining chambers and other custom accessories. We next validated the cytocompatibility of these devices by demonstrating culture and cardiomyocyte differentiation of human induced pluripotent stem cells (iPSC). Finally, we demonstrated the utility of thermoformed PC for creating 3D engineered tissues (cardiospheres). These results establish DLP-assisted PC thermoforming as a rapid, low-cost workflow for fabricating transparent, autoclavable, user-defined cell-culture accessories during prototyping and small-batch bioengineering applications.

## Introduction

Discrepancies between the way cells respond to chemical cues (e.g., drugs) and genetic mutations in cell culture versus in the body have led the biomedical science community to push toward the development of “*in vivo-*like” bioengineered platforms [1–6] in which cells are subjected to biophysical stimuli that mimic the stimuli they would encounter in the body. Many bioengineered tissue platforms rely on device-defined geometry. For example, in engineered heart tissues formed by growing cells in extracellular matrix gels stretched around PDMS posts [7,8], the distance between the posts defines engineered muscle length, prestress, and loading geometry [9]. Furthermore, the diameter of the well in which the tissue form controls overall tissue size, while chamber height and lid geometry determine working distance and access for external electrical stimulation equipment and sensors. Chamber height and geometry also affect the approach used for imaging the tissues. These parameters are often experiment-specific and may need to be adjusted across cell type, tissue size, stimulation method, imaging setup, or drug-screening format. Standard commercial plates provide robust culture surfaces but are available only in fixed geometries, while commercial spheroid or organoid plates offer limited control over well size, shape, material, and integration with external components. Therefore, the practical bottleneck comes from the unavailability of inexpensive methods for iterating custom culture accessories during device development.

For example, in the context of cardiovascular tissue engineering, neonatal rodent cardiomyocytes or human induced pluripotent stem cell (iPSC) derived cardiomyocytes (iPS-CM) are formed into organized, aligned tissues or cardiospheres using various macro- and micro-scale devices (Fig 1) [10–14]. These tissues may be further coaxed toward a more mature state via continuous mechanical [15] or electrical stimulation [16,17]. The need to provide these geometric and/or biophysical cues is challenging within conventional cell culture platforms, often requiring bespoke devices. Commercial spheroid plates are convenient but generally provide fixed well geometries and limited opportunities to tune spheroid size, array layout, accessory integration, or imaging geometry without custom manufacturing. Cost is also a limitation during iterative design, because multiple prototypes may be needed before the final geometry is selected.

**Fig 1:**
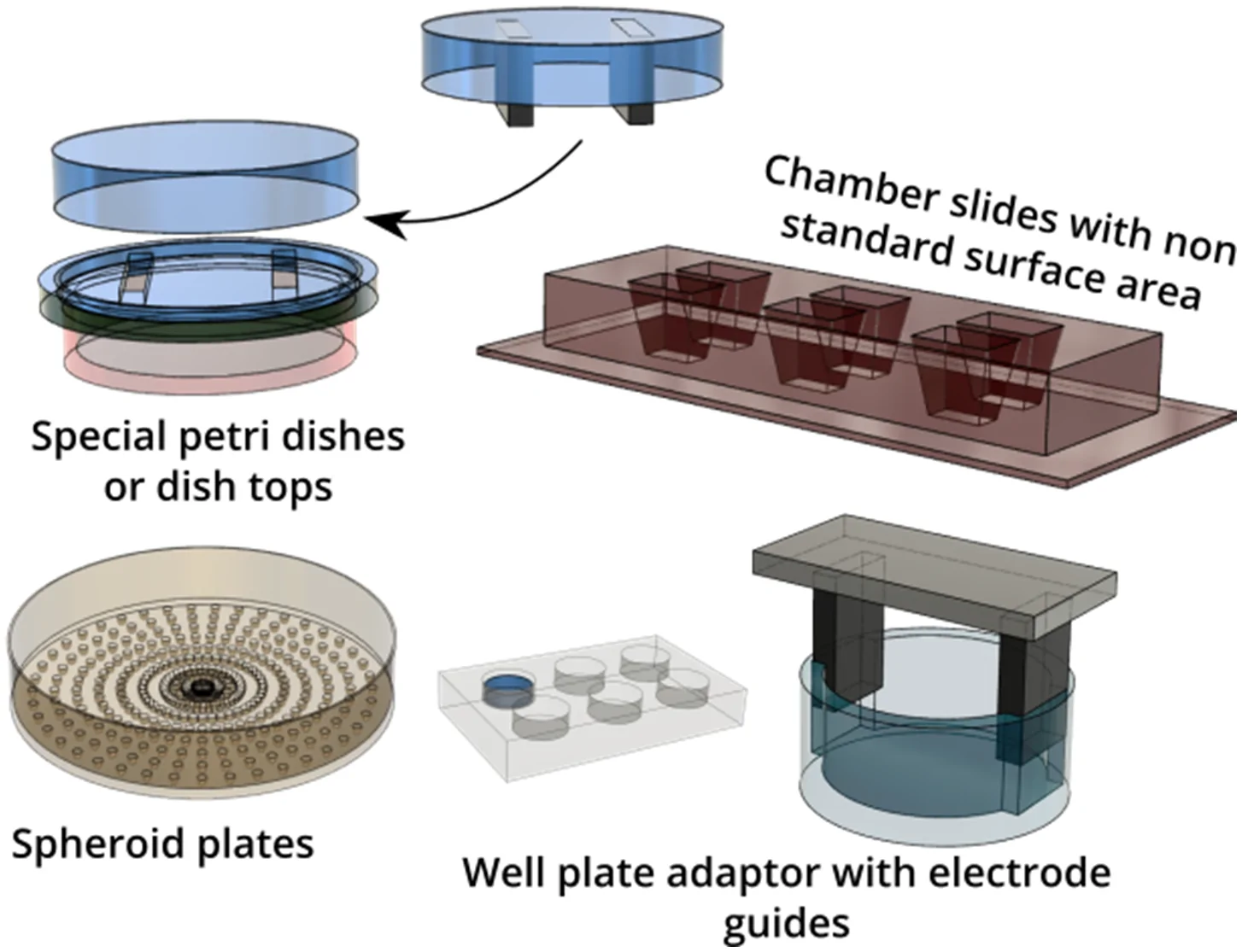
Specialized cell culture accessories are essential tools for bioengineering research: Schematic representation of customized cell culture/ tissue engineering systems used for maturing tissues with mechanical/electrical stimulation, studying tissue mechanics and live imaging with high resolution microscopy. Schematics were created using Inkscape vector graphics editor and Autodesk Fusion360.

Conventional cell culture platforms, such as well plates, culture flasks, and chamber slides, are predominantly mass-produced using polystyrene (PS) through injection molding techniques and precision-machined tooling [18]. These standardized products, while widely available and highly efficient in supporting cell growth, offer limited customization options. For example, in tissue-engineering laboratories like ours, poly(dimethylsiloxane) (PDMS) is commonly used to fabricate molds and devices that support engineered tissue formation [7,8]. These devices must be thoroughly sterilized before cell seeding and securely attached to stable support, such as glass slides or special inserts, during autoclave sterilization. Firm attachment is essential because PDMS devices that are not properly fixed may float or detach from the culture well during media changes. Such displacement can complicate imaging, reduce experimental consistency, and potentially detach the engineered tissues from the supporting posts. Direct attachment of the devices to PS culture plates is not suitable prior to autoclave sterilization, such that devices directly attached in this manner can only be disinfected with less robust means such as ethanol treatment [19].

To address the limitations of these commercial platforms, researchers commonly employ PDMS to create bespoke inserts, adaptors, and culture devices. These custom devices are made by crosslinking the PDMS elastomer over molds created through machining, 3D printing [20] or photolithography [21]. While PDMS has a long history of usage as a biocompatible material and can be easily manipulated on the micron and sub-micron scale to facilitate cell biologic studies [22], PDMS as a material presents several challenges, especially with regard to macro-scale accessories (millimeter-to-centimeter range, e.g., tissue-forming molds). First, PDMS exhibits an affinity for hydrophobic molecules, which can complicate drug screening efforts [23]. Second, for creating larger-scale devices, 3D printed molds are attractive, but many of the materials used for 3D printing leach acrylate-based monomers into PDMS. These small molecules can inhibit PDMS curing [24] and have the potential to leach out of the PDMS device during cell culture, causing toxicity [20]. Researchers can circumvent some of the challenges of using PDMS for cell culture accessories by carefully processing the 3D-printed mold surface to prevent leaching (e.g., through UV curing, thermal baking, solvent washing, silanization, applying release agents, or combining these methods) [25]. Barrier coatings such as polytetrafluoroethylene (PTFE) or parylene [26], or amphiphilic silicone formulations, can also inhibit the absorption of small molecules [27]. However, these additional processes increase the total cycle time in manufacturing, and in some cases, it may require repeated post treatments as the effect may reverse over time [28]. These limitations do not eliminate the value of PDMS, but they highlight the need for complementary fabrication methods for rigid, transparent, sterilizable, and customized cell-culture accessories.

Thermoforming is a versatile manufacturing process widely used in packaging, automotive, and dental applications [29]. In thermoforming, plastic sheets are heated until pliable, then formed over a mold using vacuum or pressure **(**Fig 2). The availability of low-cost (<$100) commercial small-scale thermoforming machines, popularized by dental guard production [30], makes this technology widely accessible. Compared to methods that require elastomer crosslinking (e.g., traditional soft lithography), the rapid nature of thermoforming allows users to generate multiple parts within a short time via repetitive use of the same mold.

**Fig 2:**
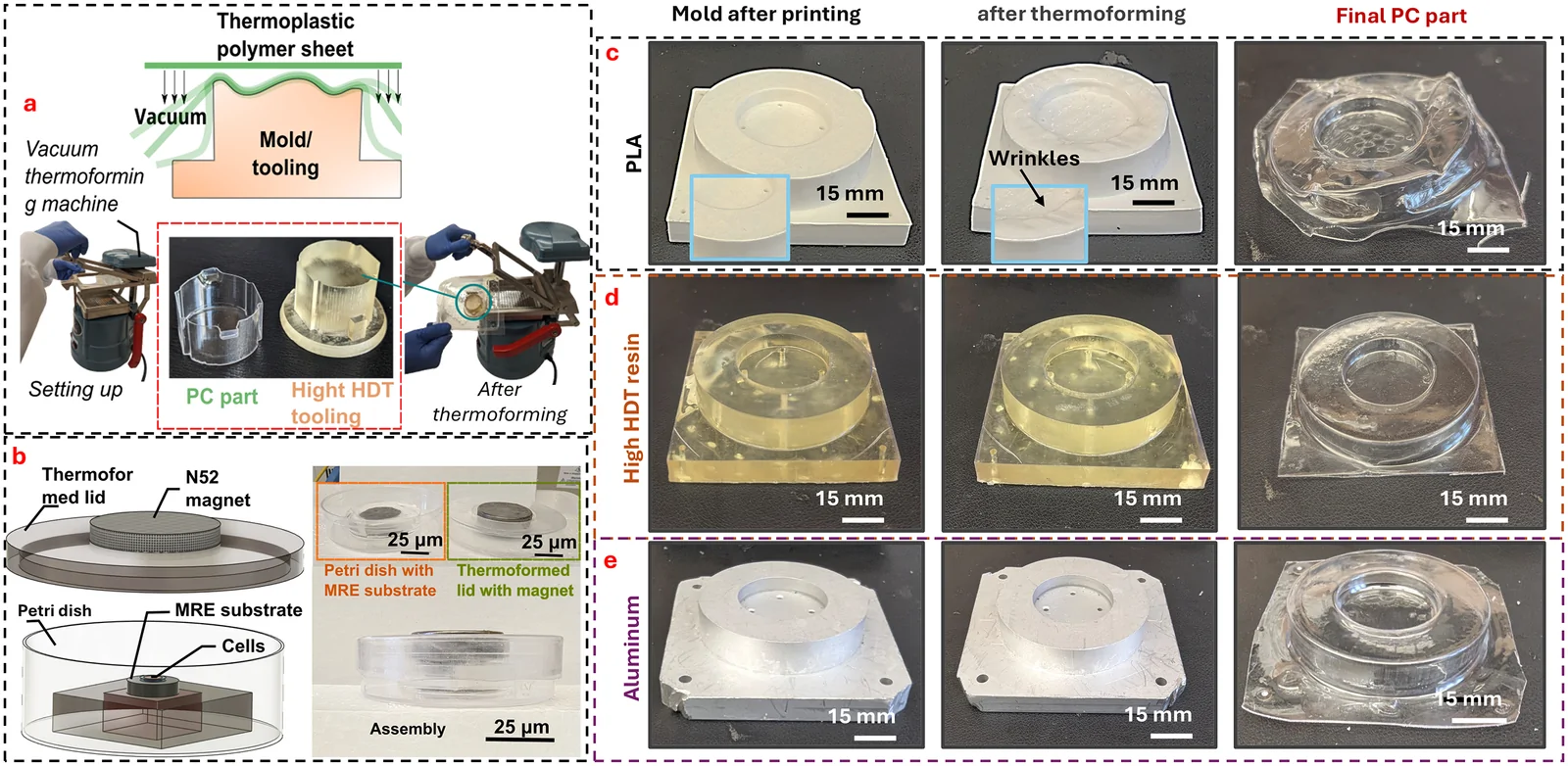
PC Thermoforming off Molds 3D-Printed from HDT Resins. **a)** Principle of thermoforming and proposed tooling method **b)** custom petri dish lid design to house permanent magnet, **c-e)** performance of different mold materials (c: poly-lactide acid, PLA; d: HDT Resin; e: 6061 aluminum) in thermoforming customized 70 mm petri dish top. Insets in panel C denote damage to the PLA mold after thermoforming.

An ideal material for thermoforming-assisted generation of cell culture devices would possess optical clarity, mechanical strength, low autofluorescence, biocompatibility, chemical inertness, and absence of harmful chemical leachates. This material would also facilitate adsorption of cell adhesive coatings (e.g., extracellular matrix proteins like fibronectin, or protein cocktails like Matrigel) [31]. Polycarbonate (PC) emerges as a viable choice among thermoformable plastics, meeting these criteria (S1 Table). Currently PC is used for microfluidic [32], tissue engineering [33], transplacental transport [34], tissue repair studies, etc. in bioengineering labs. There are many processes that exist for processing PC parts. Among them, injection molding is predominantly considered a mass production method whereas blow molding and extrusion are used for fabricating PC tubes [35]. CNC milling of PC demands post processing to restore transparency [36]. While Fused Deposition Modeling (FDM) has expanded to include high-performance engineering thermoplastics such as PC for high-impact applications, significant barriers remain for specific use cases. The high glass transition temperature (Tg) of PC necessitates specialized hardware, including nozzles capable of maintaining 300°C and high-temperature chambers (120°C-150°C) to mitigate severe warping. Even with industrial-grade equipment, FDM-processed PC often suffers from mechanical anisotropy and poor layer adhesion in thin-walled geometries, leading to fragility [37]. Furthermore, the inherent layer-by-layer deposition of FDM compromises optical transparency due to light scattering at internal interfaces. In contrast, thermoforming offers a superior alternative for fabricating thin-gauge, transparent components, as it preserves the material’s bulk structural integrity and optical clarity while remaining. A key challenge in PC thermoforming lies in selecting appropriate tooling materials and fabrication conditions. Metal molds [38] can withstand high temperatures without thermal distortion or significant surface cracking. However, machining metal molds requires special skills and expensive machinery, making it less suitable for scenarios that require frequent, iterative changes in the device design of cell culture devices (Fig 1), particularly during early prototyping stages, creates a need for alternative manufacturing methods that offer lower cost, more design flexibility.

Common materials used in FDM, like Polylactic acid, have low T_g_ (60–65°C) by design; they are thus not suitable for tooling for PC thermoforming. Even though other 3D printing techniques like stereolithography (SLA) and digital light processing (DLP) do not rely on the glass transition of the material for printing, most commonly available resin materials have T_g_ of 85°C. Recently, a specialized class of resins called high heat deflection temperature (HDT) resins has been developed for low run injection molding, automobile and dental applications [39]. HDT resins (S2 Table) can withstand temperatures ranging from 180°C to 300°C [39]. As most of these resins are stable above the glass transition temperature of PC, these resins are a good choice for the tooling material for PC thermoforming. Altogether, thermoformed PC accessories can complement PDMS-based systems by avoiding several practical limitations of PDMS accessories, including hydrophobic small-molecule absorption, limited rigidity for macroscale supports, and fabrication challenges such as curing inhibition from 3D-printed molds. Table 1 consolidates the characteristics of different methods usually used to fabricate cell culture plastic accessories and devices.

**Table 1:** Comparison of common fabrication methods used for cell culture lab applications compared to proposed thermoforming method.

Type	Remarks	Limitations	Reference
Commercial PS cultureware	Standardized, sterile, optically clear culture platforms	Fixed geometries; limited customization of inserts, lids, chambers, stimulation access, or patterned substrates	[40]
PDMS-based custom devices	Custom molds, inserts, adaptors, and soft microdevices	Hydrophobic molecule absorption; limited rigidity for macroscale supports; curing/leaching issues from 3D-printed molds	[23]
Direct 3D printing	Rapid geometric customization	Limited optical clarity, surface roughness, leachates; PC printing requires high-temperature hardware	[41]
CNC machining / metal molds	Accurate and durable parts or molds	Higher cost, specialized equipment, slower design iteration; PC may need transparency restoration	[42]
Injection molding	High-quality mass production	High tooling cost and long lead time; unsuitable for early-stage small-batch iteration	[18] S2 Table; S4 Table
Thermoformed plastic devices	Preserves optical clarity of plastic sheets and enables rapid fabrication of thin-gauge parts	Requires heat-stable tooling and optimized forming conditions	[43] S3 Table
DLP-printed high-HDT molds + PC thermoforming.	Enables rapid, low-cost, small-batch fabrication of transparent, autoclavable, user-defined PC accessories	Intended for early-stage prototyping and small-batch fabrication,	S1–S4 Tables

In this study, we demonstrate the efficacy of combining DLP printing, high HDT resins, and desktop vacuum thermoforming for rapid fabrication of custom devices, culture platforms, and accessories tailored to various studies with iPSC and iPS-CM. We anticipate this technology will support a variety of bioengineering and cell biology experiments by enabling rapid, small-batch customization of cell-culture accessories during early-stage design iteration, before designs are finalized for outsourced machining or mass production.

## Results

### High HDT tooling and preheating PC sheets help to create transparent PC inserts

We first assessed the feasibility of PC thermoforming off molds made from several candidate tooling materials: aluminum, PLA, and high HDT resin. For these studies, we chose to replicate custom designed lids for a commercially available 70 mm petri dish (Corning). Such lids find application in studies where external accessories must be placed close to the cell culture surface. For example, in our prior work [15], magnets were placed close to the surface of magnetically-responsive PDMS substrates to elicit dynamic changes in substrate elasticity, while imaging through the bottom of the cell culture surface with an inverted microscope. This required a custom lid, as standard well plate lids create too large a gap between the magnet and the substrate. For this application to be feasible, the custom lid must fit precisely above the well surface, allowing gas exchange without excessive evaporation.

PLA molds failed to keep shape integrity during thermoforming, compromising the dimensional accuracy of the PC part. In contrast, machined aluminum and high HDT molds both withstood high temperature cycles without any visible thermal distortion, leading to production of nearly identical PC devices (Fig 2). Subsequent studies confirmed that among available DLP resins, only high HDT resins could produce molds that could withstand many cycles of PC thermoforming without accumulating defects in the mold (S1 Fig). A Comparison of the mechanical and thermal properties of different DLP resins are listed in supplementary table 1.

Like many other thermoplastics, PC is hygroscopic [44] and absorbs moisture even with the relatively low humidity levels found in typical lab environments. At high temperature, these water molecules inside the PC expand, causing bubbles to form while thermoforming. We therefore tested whether preheating PC sheets before thermoforming would reduce bubble formation. Without preheating, both 0.03″ and 0.04″ PC sheets showed visible bubble formation after thermoforming, with the effect being more pronounced in the thicker 0.04″ sheets (Fig 3a, 3c, 3d). In contrast, optimized preheating substantially reduced the bubble-covered area in both sheet thicknesses, producing optically clearer thermoformed parts (Fig 3b–3d). The required preheating duration depended on sheet thickness: 0.03″ PC sheets could be thermoformed without visible bubbling after 1.5 h of preheating at 60°C, whereas 0.04″ sheets required 5–6 h of preheating. PC sheets thinner than 0.02″ could be thermoformed without bubbling even without preheating. These results indicate that the optimized preheating condition should be selected based on sheet thickness, consistent with slower heat and moisture transport in thicker PC sheets. PC sheets thickness less than 0.02″ could be successfully thermoformed without bubbling without any preheating. This likely reflects the ability of these thinner sheets to heat rapidly enough during the thermoforming process to evaporate any surface moisture before the PC itself becomes pliable.

**Fig 3:**
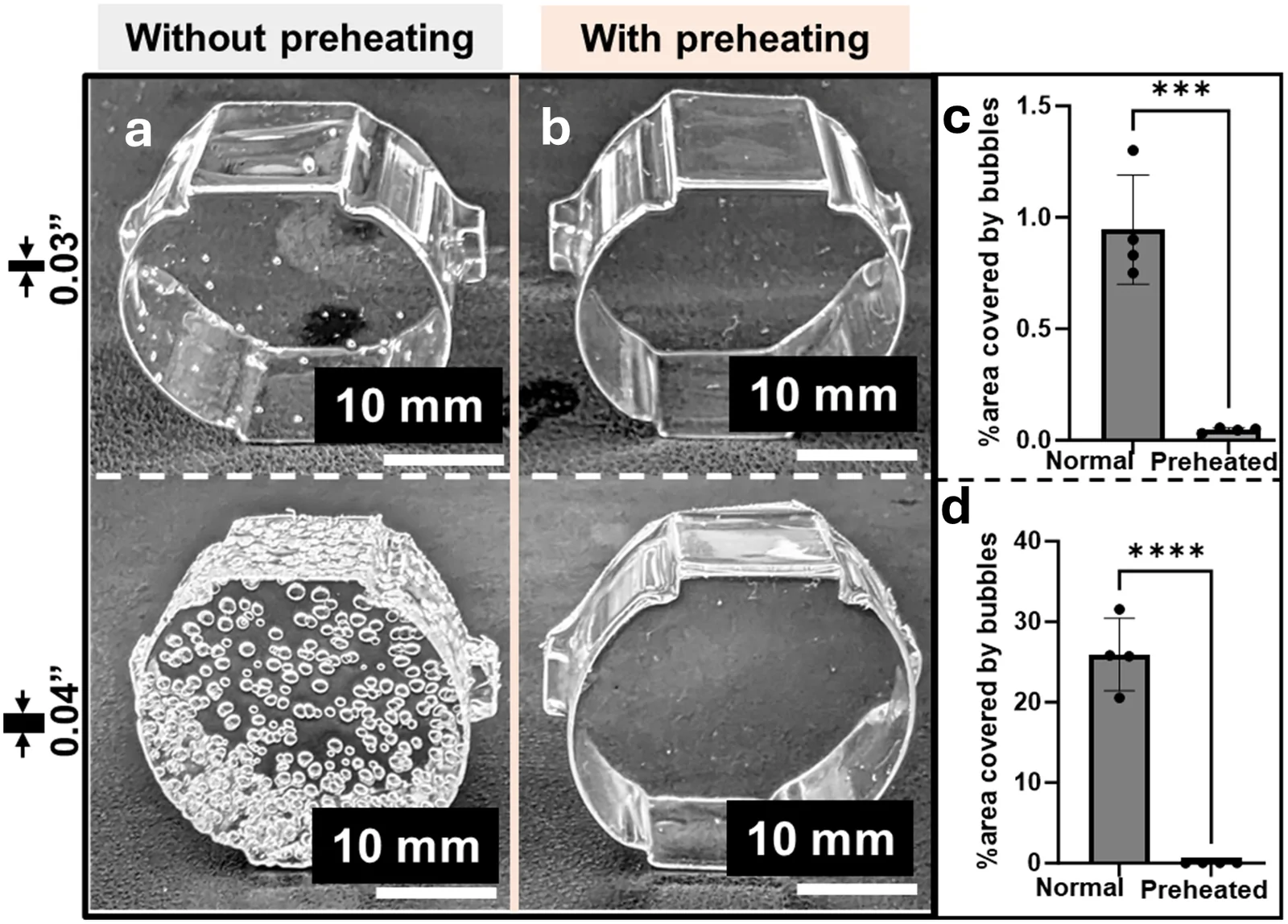
Preheating PC eliminates bubble formation during thermoforming. **a, b)** Representative images of PC inserts with different thickness that were thermoformed either a) without or b) with pre-heating for an optimized timeframe. **c, d)** Quantification of the percentage area of the final thermoformed insert covered with bubbles when thermoforming was done either with or without preheating the PC. Each datapoint represents one thermoformed device. Error bars: *SD*, *** p < 10^−2^, **** *p* < 10^−3^.

### Thermoformed PC retains optical transparency and expected resistance to hydrophobic molecule absorption

One of the essential characteristics of plastic materials used in bioengineering studies is optical transparency to facilitate high-resolution imaging [45]. Like well-inserts made from PDMS, PC-based well-inserts had similar optical properties to standard tissue culture polystyrene (PS; Fig 4a, 4b). Therefore, we next confirmed that these expected advantages of PC were retained after thermoforming. As expected [46], the hydrophobic probe molecule Rhodamine 6G absorbed into PDMS inserts (Fig 4c, 4d). This led to a strong fluorescence emission peak at 560nm. In contrast, PC inserts did not absorb Rhodamine 6G, leading to negligible changes in optical transparency and fluorescence emission after applying this probe compound (Fig 4c, 4d, 4e). This gives the validation that thermoforming did not affect the optical properties of PC.

**Fig 4:**
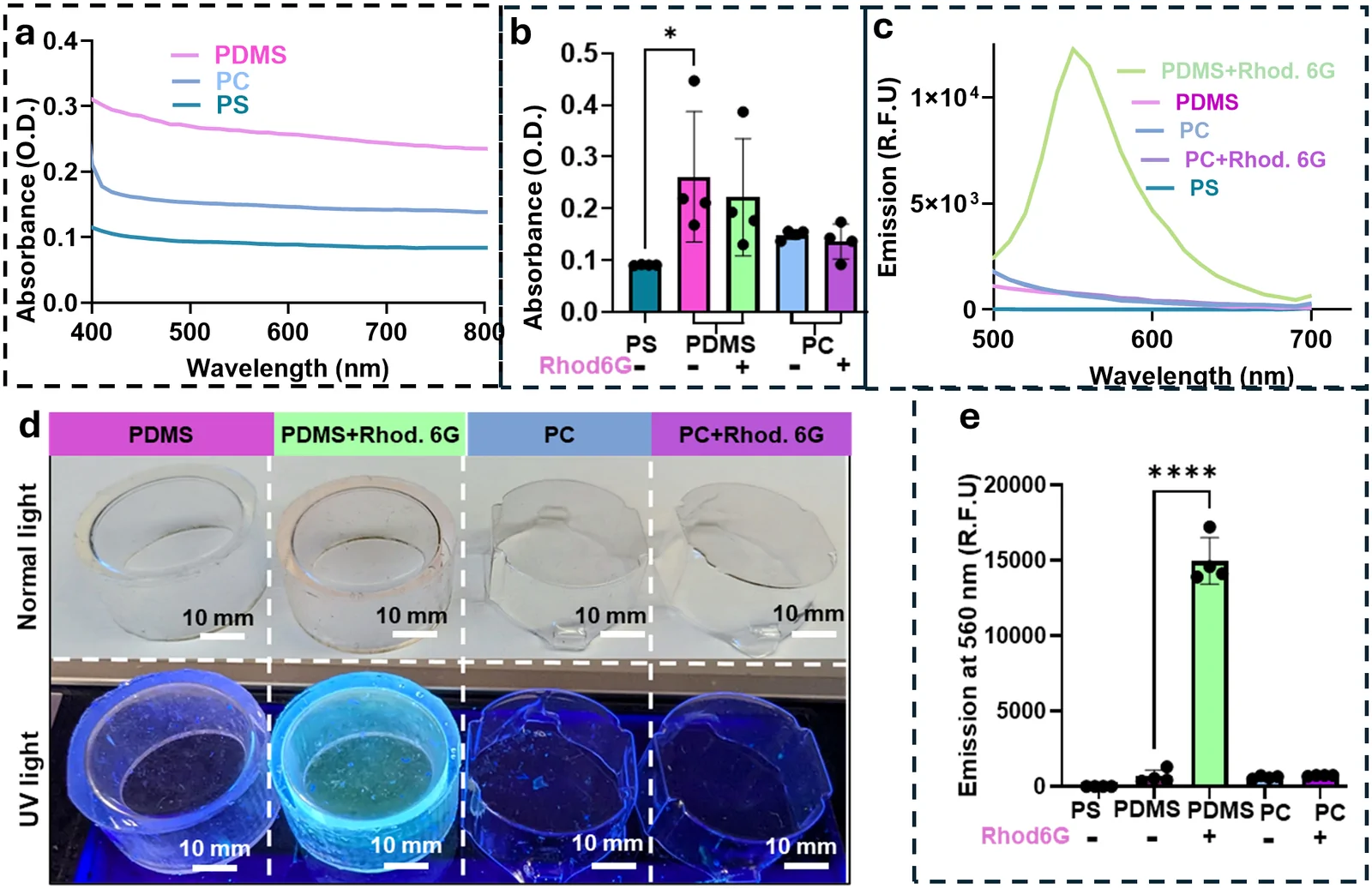
Thermoformed PC devices are optically transparent and resist absorption of hydrophobic molecules. **a)** Optical absorbance spectra of PDMS, PC and PS substrates of equivalent thickness (750 µm). **b, c)** Analysis of absorption of rhodamine G into the substrates through measurements of b) absorbance at 560nm (absorption peak of rhodamine G) or **c)** fluorescence emission spectrum after excitation at 490nm for PS, PDMS and PC either with or without pre-exposure to (1 mM) solution of rhodamine G. **d)** Representative images of molded PDMS devices, or thermoformed PC devices, either before or after exposure to 1mM rhodamine G solution. Devices were illuminated with UV light (365 nm). **e)** Quantification of peak fluorescence emission for rhodamine G (560nm) in PC and PDMS devices exposed to rhodamine G solution. Scale bars: 10 mm. Each data point represents one device. Error bars: *SD*. * *p* < 0.05, **** *p* < 10^−4^, Holm–Šidák test following global ANOVA.

### Thermoformed PC substrate supports cell culture

For PC to be used a cell culture substrate, it must facilitate robust cell adhesion and growth. Prior studies noted potential challenges in cell adhesion to PC substrates [47]. Consistent with these prior reports, we found that although C2C12 myoblasts cultured on PC substrate and tissue culture polystyrene were morphologically similar (Fig 5a), overall cell attachment was lower on PC substrates. This may reflect issues in adsorption of bioactive vitronectin, one of the main extracellular matrix proteins of cell culture serum that facilitates cell adhesion [48], onto the PC. We also noted challenges in C2C12 adhesion to uncoated PDMS, consistent with prior reports that this material requires changes in surface chemistry to facilitate robust cell adhesion [49]. In contrast, C2C12 adhesion to PC substrates pre-coated with Geltrex, a commercial protein cocktail that mimics Matrigel (where the main extracellular matrix component is laminin) [50–52] was indistinguishable from C2C12 adhesion to Geltrex-coated PS (Fig 5b).

**Fig 5:**
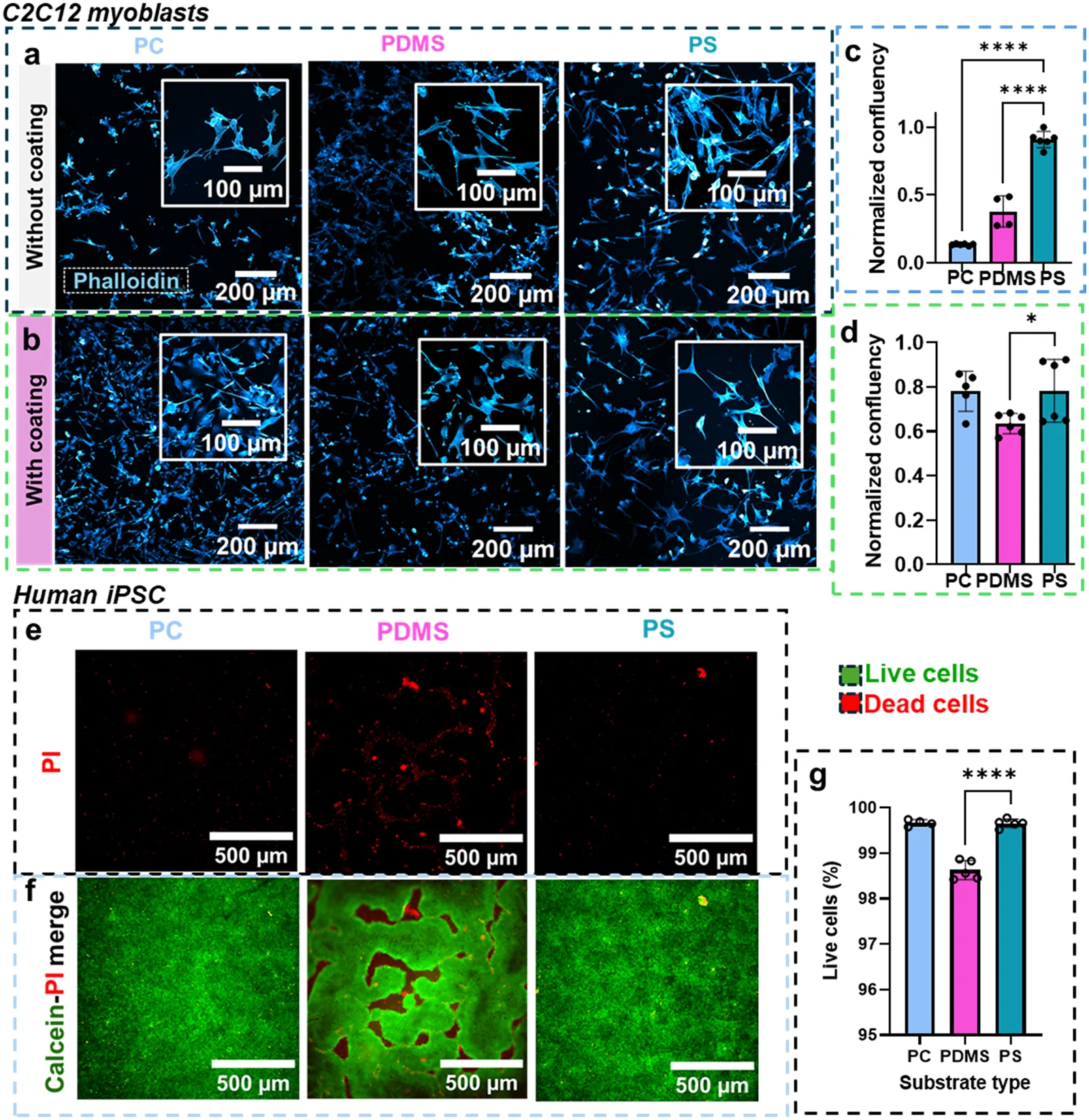
Extracellular matrix coated PC supports robust adhesion of myoblasts and induced pluripotent stem cells. **a, b)** Representative micrographs of C2C12 myoblasts when cultured on PC, PDMS and PS either without or b) with a coating of 9.47 µg/cm^2^ Geltrex^TM^. **c, d)** Quantification of overall cell adhesion (relative confluency, or area occupied by cells) of C2C12 on PS, PC or PDMS either c) without or d) with Geltrex coating. **e, f)** Representative e) calcein-AM (green) and propidium iodide (PI; red) co-staining and f) PI staining alone, for human iPSC cultured on Geltrex coated PS, PC or PDMS. **g)** Quantification of the area of live cells. Error bars: *SD*. Each data point represents one ROI. Error bars: *SD*. * *p* < 0.05, **** *p* < 10^−4^, Holm–Šidák test following global ANOVA.

While immortalized cells like C2C12 myoblasts are typically amenable to culture on a variety of different materials, pluripotent cells like iPSC typically have more stringent requirements in terms of culture substrate. For example, prior work shows that Matrigel coated tissue culture polystyrene, but not glass, supports robust human pluripotent stem cell attachment and growth [51]. We thus tested the feasibility of using Geltrex coated PC for iPSC culture. Interestingly, we observed no qualitative changes in iPSC colony morphology or viability when these cells were cultured on Geltrex coated PC or PS (Fig 5b). As expected for a material long used for cell culture, PDMS likewise supported C2C12 and iPSC culture after Geltrex coating.

### Thermoformed PC inserts support human stem cell differentiation

iPSC differentiation to cardiomyocytes (iPS-CM) is a demanding application for cell culture materials [53–55] as cells undergo dramatic changes in morphology and adhesion to their culture substrate during this process. Moreover, as with any differentiation pathway, successful differentiation of iPSC down a cardiomyocyte lineage demonstrates the ability of the culture system to support these cells in their pluripotent state. We thus tested the ability of PC inserts to support this differentiation process. In this feasibility experiment, iPSC-CM sheets differentiated on Geltrex-coated PC displayed organized sarcomeric α-actinin staining, spontaneous calcium transients, and contractile motion profiles qualitatively comparable to those observed on standard PS substrates (Fig 6a-6c).

**Fig 6:**
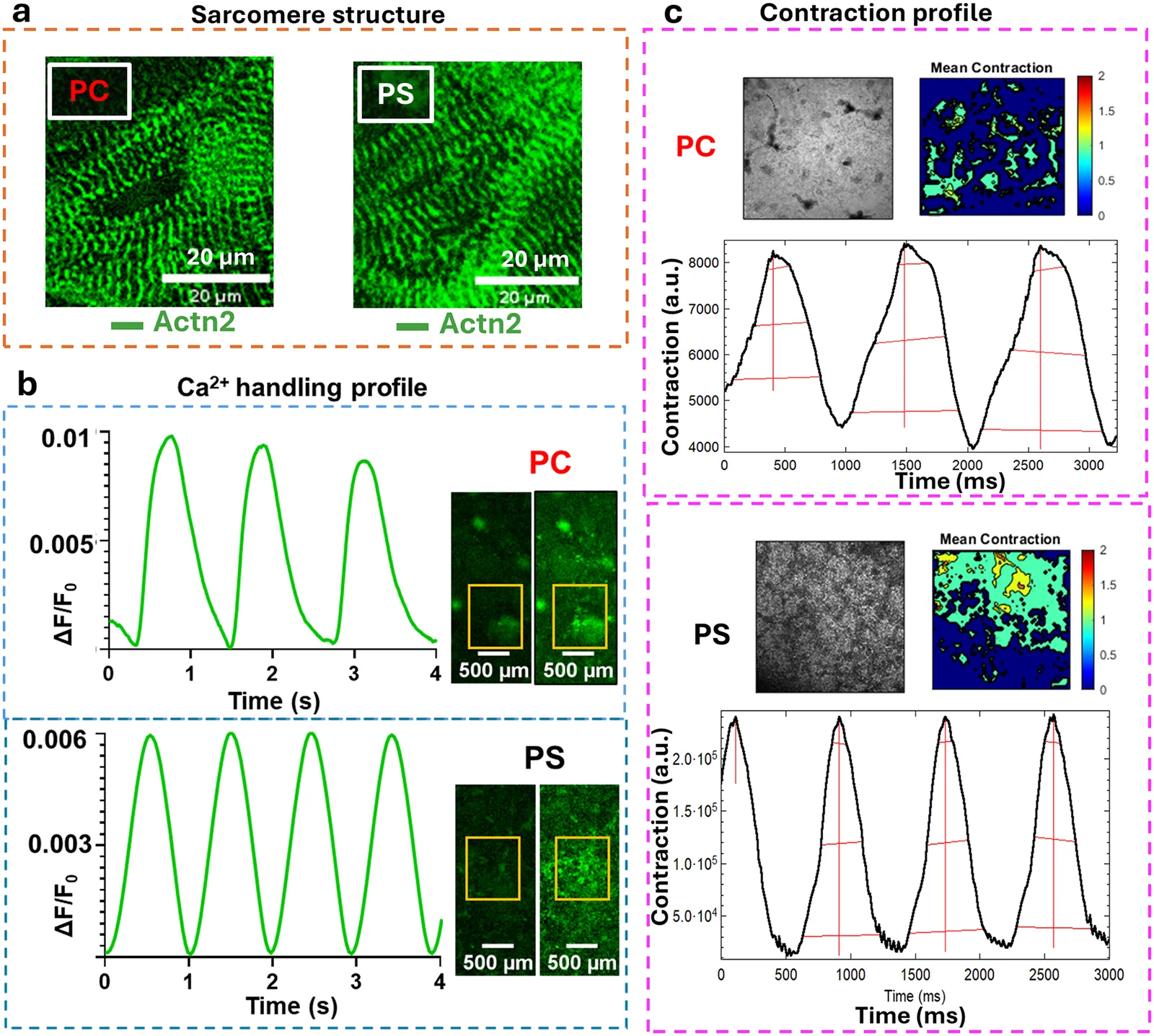
Geltrex-coated PC substrates support iPSC-cardiomyocyte differentiation. **a-c)** Representative a) micrographs of sarcomere organization, b) Ca^2+^ transient profiles and C) contractile motion profiles in iPS-CM sheets differentiated on (top) PC inserts or on (bottom) standard PS substrates. Insets in b) depict the GCaMP6f reporter in regions of the beating cardiomyocyte sheet during diastole (relaxed state, low cytoplasmic Ca^2+^) or systole (contracted state, high cytoplasmic Ca^2+^). See Supplementary video 1, 2.

3D printing can generate complex textures on the tooling material, and surface texture has marked effects on cell biology [56] Thus, we next tested the feasibility of replicating complex textures on thermoformed PC. Fig 7d shows features thermoformed with PC and PS sheets. Optical Coherence Tomography (OCT) based analysis of PS parts indicated that when parts were created using a simple dental-grade vacuum-forming machine (Fig 2) and 0.03” sheet the smallest pattern dimension that could be achieved was 1 mm (Fig 7a). However, as the dimension came closer to this limit, the shape integrity was compromised, while horizontal dimensions of such small features were relatively well preserved, vertical corners became severely filleted (Fig 7).

**Fig 7:**
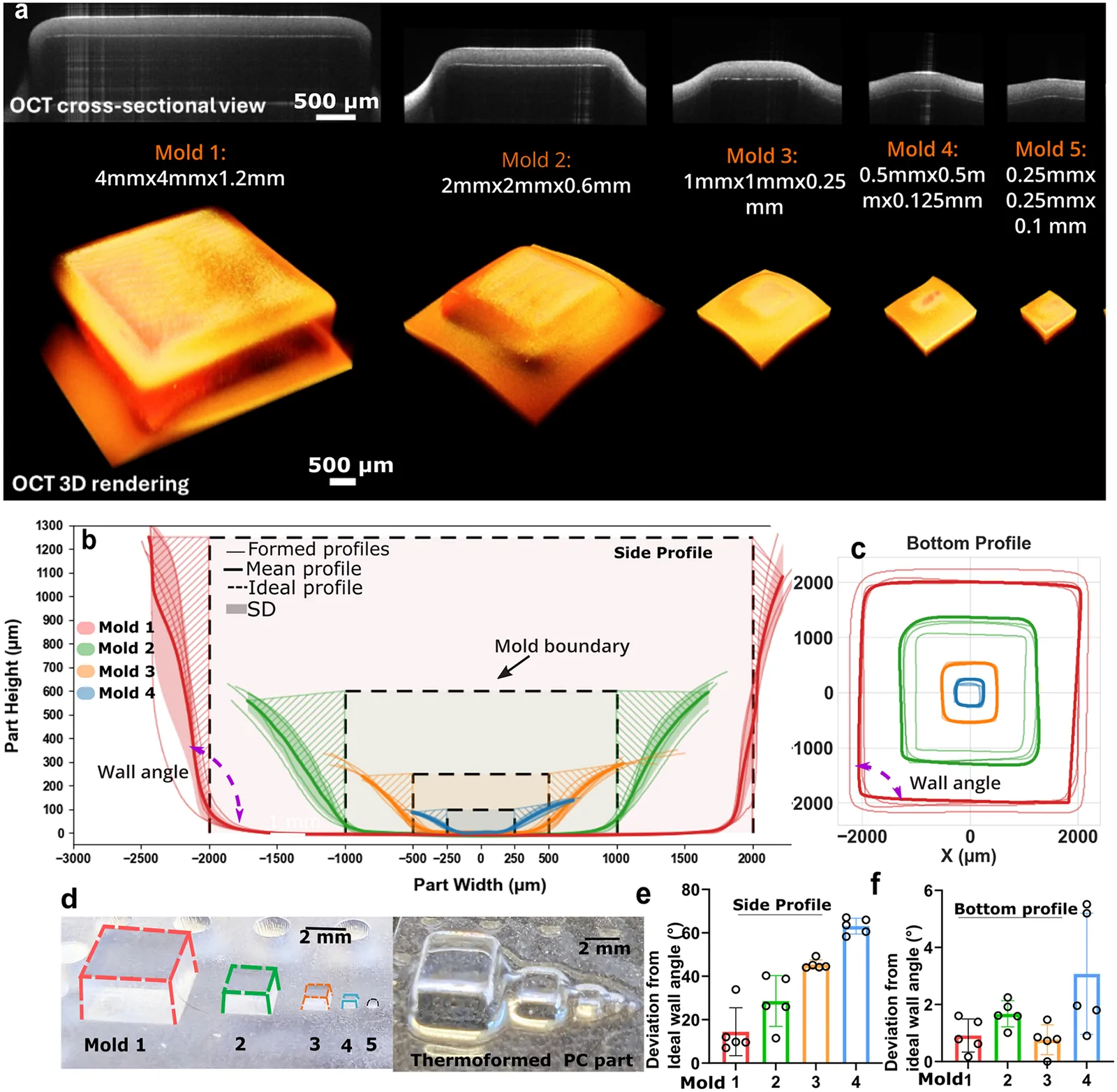
Thermoforming demonstrates good shape integrity for features in the millimeter range but has difficulty replicating mold shapes accurately when dimensions fall below 1 mm. **a)** 2D cross sectional view and 3D rendering of OCT data **b), c)** 2D form profile of b) side c) bottom **d)** Micrographs show the quality of pattern replication in PC and PS **e, f)** Quantification of deviation from ideal wall angle in e) side profile f) bottom profile.

### Thermoformed PC micro well inserts facilitate cell aggregation into spheroids

As part of the process capability demonstrations, we tried to make custom spheroid plates for forming cardioids from iPSC derived CMs. Fig 8a shows the capability of the current technique to generate features in the order of hundreds of micrometers to millimeters in PC. We have utilized this capability to make patterned inserts that can generate 3D cardiac spheroids. One of the main concerns of using iPSCs for modeling various diseases is the lower level of maturity of iPSC-derived tissue cells compared to their postnatal counterparts [2]. 3D culture either during or after the differentiation process has emerged as a promising approach to improve iPS-CM maturity. Currently, there are limited options for the culture plates for forming spheroids [14,57,58]. Using PC patterned with U-shaped microwells, we successfully patterned iPS-CM (Fig 8b) into spheroids of different sizes. Aggregates formed within 3 days of seeding and could be cultured for at least 2 weeks.

**Fig 8:**
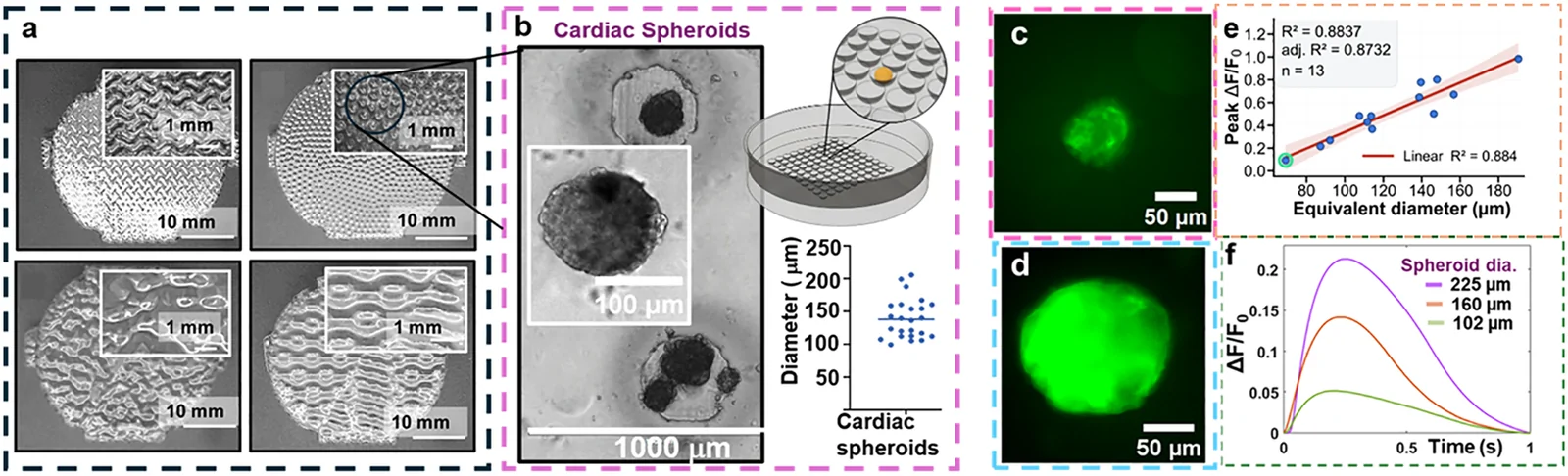
Demonstration of the capability of thermoforming to form complex patterns that can be leveraged to study cell biology. **a)** different patterns formed in PC sheets with thermoforming. **b)** cardiac spheroids formed by the hemispherical microwell pattern. **c, d)** representative images of spheroids of different sizes formed in PC captured during systole (contracted state, high cytoplasmic Ca^2+^). **e, f)**, demonstrate the difference in peak Ca² ⁺ transient amplitude (ΔF/F₀) with the change in cardiosphere size. See supplementary video 3-6.

As our prior studies suggest a strong relationship between the geometry of engineered heart muscle and iPS-CM physiology, we addressed the impact of cardiosphere geometry on calcium handling [55]. We have observed that peak Ca^2+^ transient amplitude (ΔF/F₀) increases with cardiosphere size (diameter, Fig 8c, 8d, 8f), with the two measures showing a strong positive linear correlation (R^2^ = 0.884; Fig 8e).

## Discussion

In this study, we demonstrate the feasibility of producing custom cell culture accessories from thermoformed PC that are transparent, mechanically and chemically stable, and which support cell culture and fluorescence imaging, without heavy capital investment on machineries. Optimization of PC preheating time and temperature minimized bubbling down to a negligible level, ensuring optical transparency and mechanical stability, whilst allowing user-defined patterning. This in turn allowed us to investigate the relationship between the geometry and Ca^2+^ handling of iPSC-derived cardiobodies.

As the custom cell culture accessories might be designed to support cell growth as a substrate or to “house” various subsystems inside the well keeping the environment sterile, it becomes critical to ensure that thermoformed PC parts are biocompatible. Our studies show that human iPSC, one of the more challenging cell types to maintain in culture, can be expanded and even differentiated on the PC inserts. Compared to iPS-CM differentiated on PS plates, PC inserts did not show any change in morphology or contractility (See supplementary Video). Since spheroids can be generated using commercial round-bottom and microwell platforms, this experiment demonstrates that user-defined thermoformed PC microwells can support cardiac aggregate formation and live calcium imaging, thereby validating the workflow for customized 3D culture accessories.

Vacuum thermoforming has some inherent advantages and limitations in the context of cell culture device fabrication. In terms of advantages: the aforementioned lack of absorption of small hydrophobic molecules into PC (Fig 4), rapid time-course for making devices (hundreds of devices can be produced in <1 hour from a single mold), and optical transparency of PC devices are all attractive.

In terms of disadvantages of the present approach and of the use of PC in particular: as PC has low flowability during injection molding, it is not recommended to reduce the wall thickness less than 1 mm [59]. Contrary to that, thermoforming can achieve wall thickness as low as 100 μm which makes it suitable to make attachments that support high resolution imaging. However, replicating mold designs that feature large overhangs, blind holes, or microscale texture remains challenging in thermoforming. Achieving sharp corners is also difficult (Fig 7). Second, while the rigid mechanical properties of thermoformed PC are highly advantages for handling, specific applications that require substrates with mechanical properties tuned either to control cell biology or measure contractility may require softer materials, or coating the PC materials with a thin layer of soft materials such as hydrogel or PDMS. Low oxygen permeability of PC can introduce challenges if thermoformed parts are used in closed bath culture, microfluidic devices [60] or in experiments where oxygen transfer through the substrate is essential. However, in open bath culture this low permeability is unlikely to affect cell growth, especially if, as in this study, the cultures are agitated to improve oxygen transport. Finally, we note that PC is susceptible to photodegradation during extended UV irradiation especially in the presence of oxygen [61]. Therefore, it is better to avoid UV sterilization of the PC attachments when they are used for cell culture applications. This drawback is overcome by the ability to apply autoclaving, which is more robust at eliminating microbes compared to UV. In recent studies, we exploited the ability to autoclave sterilize the PC well plate inserts for long term culture, during which we performed serial electrical stimulation on the same device to assess cardiac micro-tissue physiology at a defined beat rate [7].

In the future, it will be of interest to expand the design space for thermoformed PC devices. The upper size limit of structures made by this method is restricted by the thermoforming machine used. We note that industrial-grade machines can thermoform sheets meters wide and several inches thick, while the desktop machine we use (PlastVac p7, Bioart) holds sheets up to 5” x 5”; most tissue engineering or cell culture accessories fall within this range. However, creating large-scale arrays of such devices could be facilitated with a larger machine.

In terms of the lower limit for features achievable with PC thermoforming: The dimensional accuracy of a thermoformed part depends on several factors: the particular thermoforming method employed, the precision of the mold, the type of sheet material, and the sheet thickness. Polycarbonate sheets with a thickness of 0.03 inches typically provide good dimensional accuracy for features in the millimeter scale. For applications requiring smaller feature sizes or cell-scale patterns, it will be of interest in future studies to employ ultra-thin PC films (around 0.005″) combined with pressure forming [62] techniques to enhance feature resolution. Further refinement of our approach to allow smaller geometric patterns. Stringent control over the well dimensions may help to get fine-tuned control over organoid morphology, and may affect the 3D tissue functions in long term culture [63], in future studies.

## Conclusion

Here, we suggest a method for rapid fabrication of custom cell culture/tissue culture accessories by combining DLP printing, high HDT resins and thermoforming PC sheets. As a 0.03″ PC sheet takes 20 seconds to soften and it takes less than one minute to fabricate a part, and the mold can be reused for multiple cycles without any visible thermal damage, this creates a low-cost approach for rapidly creating large numbers of bespoke cell culture/tissue engineering accessories. Apart from the sterilizable cell culture/ tissue engineering accessories, this method can also fabricate everyday lab accessories like funnels, containers, mixing dishes, tube holders, etc. (See S2 Fig). We further demonstrate that thermoformed PC devices are capable of facilitating cell growth and fluorescent imaging. As 3D cell culture and co-culture are proving critical in many in vitro disease modeling studies [2,64] researchers can use the simple approach presented here to design, fabricate and test new customized devices to fashion tissues with bespoke geometry.

## Materials and methods

### Mold preparation and PDMS insert preparation

All the designs are made with Autodesk Fusion 360 and sliced with *CHITUBOX Basic* (Version 2.0). We used Phrozen Mighty 8K DLP printer and Siraya Tech Sculpt high HDT resin to print the thermoforming molds. Other DLP resins including Conjure rigid resin, Phrozen clear 8k resin, Phrozen gray 8k resin, TH47 tough resin are used to compare the performance of HDT resins in multiple cycles of thermoforming. An additional 3D printed mold is printed for making PDMS inserts. The PDMS inserts are prepared using Sylgard 184 and hydrogel-assisted double molding method [20] by creating a soft negative mold of the 3D print. After curing the PDMS inserts in 37°C for 24 hours, they are demolded from the soft mold and completely cured in 60°C for additional 48 hours.

### Thermoforming

We used Plastvac P7 Dental Vacuum Forming Machine (Bioart, Brazil) for thermoforming PC parts. We used RowTec® Polycarbonate Film of thickness (0.03”, 0.04”). Thickness less than 0.03” was difficult to handle and not watertight due to micro cracks during thermoforming. Thickness greater than 0.04” was difficult to remove from the mold after thermoforming. For detailed steps on thermoforming, see S1 File (Detailed Method to understand the step by step procedure we used for thermoforming).

### Bubble area quantification

Bubble-covered area was quantified from brightfield images of thermoformed inserts using a custom Python image-analysis pipeline based on OpenCV and NumPy. Images were imported in batches and processed sequentially. Each image was converted to 8-bit grayscale, and the outer boundary of the formed insert was defined manually by selecting three points along its circumference; these points were used to fit a circular region of interest. To compensate for spatial variations in illumination, bubble features were identified from the absolute local intensity deviation normalized by the local intensity standard deviation. Bubbles were then segmented using a fixed local-contrast threshold, with identical background-correction, thresholding, and morphological-processing parameters applied within each sheet-thickness comparison. Morphological closing and region filling were used to join fragmented bubble boundaries and calculate their projected area (See S3 Fig). Bubble-covered area was expressed as the total segmented bubble area divided by the area of the circular insert region of interest. Segmentation masks were visually inspected for every image and manually corrected when edge glare, shadows, or other artifacts were incorrectly included or when bubble regions were missed.

### Optical coherence tomography

A customized Spectral Domain Optical Coherence Tomography (SD-OCT) system [65,66] recorded the thermoformed accessories. In brief, a superluminescent diode (EXALOS, EXC250023−00, central wavelength: 1300 nm, spectral range: 180 nm) provided the light source of OCT. A spectrometer (Wasatch Photonics, Cobra 1300) comprising a 2048-pixel InGaAs line-scan camera (Sensors Unlimited, GL2048) allowed a 147 kHz A-scan rate at its maximum. The resolutions of the system were ~2.83 μm (transverse) and ~3.95 μm (axial) in air (refractive index = 1.0). Each 3D-OCT image covered the corresponding field of view of each mold at a pixel size of 2–4 μm at 50 μs exposure time. At ~1300 nm wavelength, the refractive index of PS was approximately 1.57 [67]. Based on this, voxel size in x-y-z dimensions was normalized isotropically using MATLAB.

### iPSC-cardiomyocyte differentiation

All iPSC experiments were conducted with Wild Type C (WTC) human hiPSC line which express single copy of GCaMP6f (Coriell Repository # GM25256). These cells undergo at least 3 passages in Essential 8 medium before starting the differentiation protocol. On all the conditions (PC, PDMS, and PS), culture surface was coated with Geltrex™ (Thermo Fisher Scientific, Waltham, MA) diluted in KnockOut™ DMEM (Thermo Fisher Scientific) at a ratio of 1: 100 to ensure cell attachment. During first 4 days of differentiation, the cells were cultured in RPMI 1640 (Thermo Fisher Scientific) with B27 supplement without insulin and with 150 μg/mL L-Ascorbic acid (Sigma, Millipore Sigma, St. Louis, MO). Intermittently, additional reagents were supplied to guide them to cardiac lineage. After ensuring 75−80% confluency, the cells were treated with 6 μM CHIR99021 (Biogems) for GSK-3β inhibition. Wnt signaling inhibitor (5 μM IWP-2, Biogems) was supplied after 48 hours. On day 4, IWP-2 was removed, and the medium contained only R− and L-ascorbic acid. Beginning on day 6, the cells were maintained in RPMI 1640 supplemented with B-27 containing insulin (R+), with media refreshed on days 6, 8, and 11, and then every three days. Beating activity in the cell layer typically began to emerge between days 6 and 10.

For making cardiobodies, the cells are further replated and purified. On day 15 of differentiation, contracting cardiomyocyte layers were enzymatically dissociated using dispase and 1 × TrypLE^TM^ Express (1X) for 15 minutes each. Cells were carefully pipetted in knockout DMEM containing 20% fetal bovine serum (Gibco) to achieve a single-cell suspension. The cells were then resuspended in RPMI 1640 medium supplemented with B27 (with insulin) and 10 μM Y27632 and transferred to Matrigel-coated plates. Beating activity typically reappeared within 24 hours. On day 16, the medium was refreshed with standard RPMI/B27. For metabolic purification, between days 20 and 24, cells were cultured in glucose-free medium supplemented with 4 mM sodium L-lactate (Sigma), promoting selective survival of cardiomyocytes. Cultures were then transitioned back to RPMI/B27 over the following 10 days, gradually increasing the proportion of RPMI/B27 to 100% over the final 3 days. The cells are used for cardiobodies within 3days after transition.

### C2C12 immortalized mouse myoblast culture

C2C12 myoblasts were seeded onto various surfaces at a density of 5,000 cells per cm^2^ using Dulbecco’s Modified Eagle Medium (DMEM; Thermo Fisher Scientific) supplemented with 1% fetal bovine serum (FBS; Thermo Fisher Scientific).

### Cell viability

Cell viability was evaluated using Calcein-AM and Propidium Iodide (PI) staining (Live/Dead Imaging Kit, Thermo Scientific). Cells were incubated with 2 μM Calcein-AM and 4 μM PI in PBS at 37 °C for 30 minutes, protected from light. After washing with PBS, fluorescence imaging was performed using a fluorescence microscope. Live cells fluoresced green (Calcein-AM), and dead cells fluoresced red (PI). Quantification was done using ImageJ.

### Cardiosphere formation

Purified cardiomyocytes are dissociated using TrypLE^TM^ Select 10X and are related on to the special PC inserts with RPMI 1640 medium supplemented with B27 (with insulin), 10% FBS and 10 μM Y27632. The inserts are centrifuged at 100 rpf for 4 minutes to allow the cells to aggregate to single microwells. The inserts are carefully transferred to the incubator and kept for 48 hours. The media changed carefully to RPMI 1640 medium supplemented with B27 (with insulin) after 48 hours. Videos are captured after culturing cardiobodies for 5 days.

### Calcium transient measurement

Intracellular calcium activity was monitored using the GCaMP6f calcium indicator. Time-lapse fluorescence imaging was performed at a frame rate of 100 fps to capture calcium transients. A custom MATLAB script [68] was used to isolate individual calcium waveforms and quantify key parameters, including spontaneous beating frequency, signal amplitude, rise time, and decay kinetics.

### Contraction analysis

The contraction maps were generated using MotionGUI [69] software and the contraction profile graphs were plotted using MUSCLEMOTION [70].

### Immunostaining

Cardiomyocyte monolayers were fixed with 4% paraformaldehyde (PFA) for 2 hours at room temperature, followed by PBS washes. Cells were permeabilized using 0.1% Triton X-100 for 10 minutes, then blocked for 45 minutes in a solution containing 3% goat serum and 3% BSA in 0.1% Triton X-100. Samples were incubated with a primary antibody against sarcomeric α-actinin (ACTN2) and subsequently imaged using a confocal microscope (Olympus Fluoview FV1200, Tokyo, Japan).

### Statistical analysis

All data are presented as mean ± standard deviation (SD). For comparisons between two groups, statistical significance was assessed using unpaired two-tailed Student’s t-tests. For comparisons involving more than two groups, one-way ANOVA followed by Holm–Šidák post hoc tests was used to correct for multiple comparisons. A minimum of four biological replicates (independent inserts) were used for each condition. Statistical significance was defined as p < 0.05.

## Supporting information

S1 Table Mechanical and chemical properties of tissue culture polystyrene, PDMS and PC. (S1 Table.DOCX)

S2 Table Comparison of methods used for processing PC. (DOCX)

S3 Table Mechanical and thermal properties of mold materials. (DOCX)

S4 Table Comparison of processing cost for PC inserts with different fabrication methods. (DOCX)

S1 Fig Extended analysis of the impact of tooling material composition on thermal deformation resistance. a) Shows molds fabricated with different DLP resins, PLA and aluminum b) common thermal defects on the mold after being exposed to high temperature. Scale bar: 10 mm.
(PNG)

S2 Fig Lab accessories thermoformed using desktop vacuum thermoforming machine. (PNG)

S3 Fig Steps in bubble area analysis. (PNG)

S1 File Detailed method steps in PC thermoforming using high HDT resins. (DOCX)

S1 Video Depiction of graphite electrode placement into the PC insert. (MP4)

S2 Video Beating cardiomyocytes derived from iPSC in a commercial PS well plate. (GIF)

S3 Video Beating cardiomyocytes derived from iPSC in a PC insert. (GIF)

S4 Video Brightfield video of cardiobodies formed in a PC insert. (GIF)

S5 Video Fluorescent video of GCaMP6f signal in cardiobodies formed in a PC insert. (GIF)
