# Supplementary material for: Rapid thermoforming of polycarbonate cell culture accessories from 3D printed molds
Source: PLoS One. 2026 Oct 26;21(10):e0361065. doi: 10.1371/journal.pone.0361065 (PMC13704808; doi:10.1371/journal.pone.0361065)
Supplement: Supplementary file 1 [file pone.0361065.s001.docx]

| **S1 Table Mechanical and chemical properties of tissue culture polystyrene, PDMS and PC**[1], [2] | | | |
| --- | --- | --- | --- |
| Properties | Atactic Polystyrene (tissue culture polystyrene) | PDMS  (Sylgard 184, 10:1) | PC |
| Fabrication process | Injection molding | Casting | Injection molding  Thermoforming |
| Refractive index | 1.59 | 1.4348 | 1.584 |
| Contact angle (°) | 80 | 110 | 90 |
| Abbe number | 30-34 | 40 | 30 |
| Autofluorescence | Low | Low | Usually low, strong autofluorescence at shorter wavelengths, (300-400 nm) |
| Glass transition temperature (°C) | 60-100 | n/a | 147 |
| Young’s modulus | 3.4 GPa | 1.74 MPa | 2.3-4.1 GPa |
| Incompatible solvents | Poor resistance to aromatic and chlorinated hydrocarbons, esters, and ketones | Swells in many organic solvents including Hexane, Toluene | Methyl alcohol, acetone, ketones, ethers, aromatic and chlorinated hydrocarbons, aqueous or alcoholic alkaline solutions. |

References

[1]	P. Painter, M. Sobkowiak, and Y. Park, “Vibrational Relaxation in Atactic Polystyrene:  A Calculation of the Frequency Correlation Functions of Ring Stretching Modes and Their Variation with Temperature,” *Macromolecules*, vol. 40, no. 5, pp. 1738–1745, Mar. 2007, doi: 10.1021/ma062423c.

[2]	“McMaster-Carr.” Accessed: Apr. 30, 2025. [Online]. Available: https://www.mcmaster.com/
