# Supplementary material for: Rapid thermoforming of polycarbonate cell culture accessories from 3D printed molds
Source: PLoS One. 2026 Oct 26;21(10):e0361065. doi: 10.1371/journal.pone.0361065 (PMC13704808; doi:10.1371/journal.pone.0361065)
Supplement: Supplementary file 2 [file pone.0361065.s002.docx]

| **S2 Table Comparison of methods used for processing PC**[3], [4]**,** [5] | | | |
| --- | --- | --- | --- |
| Technology | Optical Clarity | Cell Compatibility | Prototyping Cost & Speed |
| Standard FDM | *Not usable for PC* | | |
| High-Temp FDM | Low, Light scattering at the layer interfaces reduces transparency | Compatible with cell culture | High machine cost. Low production cost. Fast process. Limited by part fragility and high warping. |
| PC-like Resin (SLA/DLP) | Transparency is not adequate for high magnification imaging | Lower compatibility due to the presence of photo initiators | Moderate Cost. Fast. |
| Injection Molding | Excellent clarity | High cell compatibility | Mass production process. High cost for low volume prototyping. Tooling can be expensive. Low flowability of PC will restrict the minimum wall thickness |
| Thermoforming | Excellent clarity. Preserves original optical-grade sheet finish. | High cell compatibility | Low-cost parts. Fast process. |

References

[3]	“Fast Hydrolytically Degradable 3D Printed Object Based on Aliphatic Polycarbonate Thiol‐Yne Photoresins - Wu - 2021 - Macromolecular Chemistry and Physics - Wiley Online Library.” Accessed: Apr. 27, 2026. [Online]. Available: https://onlinelibrary.wiley.com/doi/full/10.1002/macp.202000435

[4]	B. Srinivas, P. Satishkumar, R. Saminathan, and B. Srikanth, “Optimization of process parameters in 3D printing of polycarbonate for high heat applications,” *Interactions*, vol. 245, no. 1, p. 119, Jun. 2024, doi: 10.1007/s10751-024-01961-7.

[5]	U. A. Dar, Y. J. Xu, S. M. Zakir, and M.-U. Saeed, “The effect of injection molding process parameters on mechanical and fracture behavior of polycarbonate polymer,” *J. Appl. Polym. Sci.*, vol. 134, no. 7, 2017, doi: 10.1002/app.44474.
