# Supplementary material for: Rapid thermoforming of polycarbonate cell culture accessories from 3D printed molds
Source: PLoS One. 2026 Oct 26;21(10):e0361065. doi: 10.1371/journal.pone.0361065 (PMC13704808; doi:10.1371/journal.pone.0361065)
Supplement: Supplementary file 3 [file pone.0361065.s003.docx]

| **S3 Table Mechanical and thermal properties of mold materials**[6], [7], [8], [9] | | | | | | | |
| --- | --- | --- | --- | --- | --- | --- | --- |
| Property | Conjure Rigid Gray | Phrozen Aqua Clear | Phrozen Aqua Gray | TH72 | Siraya Sculpt High HDT | PLA (Polylactic Acid) | Aluminum |
| Type | DLP Resin | DLP Resin | DLP Resin | DLP Resin | DLP Resin | Thermoplastic | Metal |
| Ultimate tensile Strength (MPa) | 39.13 | 17.6 | 17.6 | 26.23 | 35 | 40 | 310 |
| Heat Deflection Temperature (°C) | 70 | 70 | 70 | 80 | 180 | 60 | 500+ |
| Shore Hardness | 85–90D | 70D | 70D | 75D | 90D | Not applicable | Not applicable |
| Transparency | Opaque | Clear | Opaque | Opaque | Clear | Opaque | Opaque (metallic) |

References

[6]	“ASM Material Data Sheet.” Accessed: Apr. 29, 2025. [Online]. Available: https://asm.matweb.com/search/specificmaterial.asp?bassnum=ma6061t6

[7]	“Pushing the Boundaries: How Our High-Temp Resins are Transforming 3D Printing,” Siraya Tech. Accessed: Feb. 09, 2025. [Online]. Available: https://siraya.tech/blogs/news/pushing-the-boundaries-how-our-high-temp-resins-are-transforming-3d-printing

[8]	“Ultimate 3D Printing Material Properties Table.” Accessed: Apr. 29, 2025. [Online]. Available: https://www.simplify3d.com/resources/materials-guide/properties-table/

[9]	The4, “Rigid Resin,” ChituSystems. Accessed: Apr. 29, 2025. [Online]. Available: https://www.chitusystems.com/products/rigid-resin
