# Supplementary material for: Rapid thermoforming of polycarbonate cell culture accessories from 3D printed molds
Source: PLoS One. 2026 Oct 26;21(10):e0361065. doi: 10.1371/journal.pone.0361065 (PMC13704808; doi:10.1371/journal.pone.0361065)
Supplement: Supplementary file 4 [file pone.0361065.s004.docx]

**S4 Table Comparison of processing cost for PC inserts with different fabrication methods**[10], [11]

| Fabrication method | Tooling cost | Per part cost | Total cost for 50 parts | Lead time for 50 parts | Design flexibility |
| --- | --- | --- | --- | --- | --- |
| DLP printing+ Thermoforming | $0.65-0.9 | $0.48-0.60 | $24.65-30.9 | 1 day | High |
| CNC milling+ Thermoforming | $142-347 | $0.48 -0.60 | $166.00-377 | 3-5 days | Low |
| Metal binder jet printing+ Thermoforming | $205-350 | $0.48-0.60 | $229.00-380 | 3-5 days | Moderate but expensive |
| Direct metal laser sintering+ Thermoforming | $164-469 | $0.48-0.60 | $188.00-499 | 3-5 days | Moderate but expensive |
| Injection molding | $4580-8500 | $27.78-35.5 | $9,564.00-10275 | 3-6 weeks | Very low |
| Direct PC printing | $0 | $62-75 | $3,100.00-3750 | 1-3 days | High |

References

[10]	“Custom Manufacturing on Demand | Production Parts and Prototypes | Xometry.” Accessed: Apr. 29, 2026. [Online]. Available: https://www.xometry.com/

[11]	“Protolabs | Rapid Prototyping & On-demand Production Services.” Accessed: Apr. 29, 2026. [Online]. Available: https://www.protolabs.com/
