# Supplementary material for: Rapid thermoforming of polycarbonate cell culture accessories from 3D printed molds
Source: PLoS One. 2026 Oct 26;21(10):e0361065. doi: 10.1371/journal.pone.0361065 (PMC13704808; doi:10.1371/journal.pone.0361065)
Supplement: Supplementary file 5 [file pone.0361065.s008.docx]

**S1 Detailed Method** Steps in PC thermoforming using high HDT resins

Items needed:

1. Desktop vacuum thermoforming machine (e.g. [Amazon.com: Plastvac P7 110V Dental Vacuum Forming Machine, Laboratory Heat Molding Equipment, Made in Brazil, Sold by JMU : Industrial & Scientific](https://www.amazon.com/Plastvac-Forming-Machine-Laboratory-Equipment/dp/B09CYGSB2T))
2. Polycarbonate sheets of desired thickness ([film | McMaster-Carr](https://www.mcmaster.com/products/polycarbonate-film/))
3. DLP printer with good resolution (e.g. [Phrozen Sonic Mighty 8K Resin 3D Printer | Phrozen Technology: Resin 3D Printer Manufacturer](https://phrozen3d.com/products/sonic-mighty-8k))
4. High HDT resin (e.g. [Sculpt High-Temperature Resistant | Resin for 3D Printing](https://siraya.tech/products/sculpt-resin-high-temperature-resistant-resin))
5. Preheating oven
6. Heavy duty scissors

Steps:

1. Prepare the DLP-printed mold, ensuring sufficient draft angle for easy demolding. After washing and curing, bake the mold at 60 °C for 1 hour.
2. Preheat the PC sheets. For sheets thinner than 0.02”, preheating may be skipped; for thicker sheets, preheat at least 1 hour to prevent bubbling.
3. Cut the PC sheets to dimensions that fit the fixture.
4. Switch on the heating unit and monitor the sheets as they soften and begin to sag. If bubbling occurs, preheat the sheets for a longer duration.
5. Always wear heat-resistant gloves when handling hot PC sheets.
6. Place the high HDT mold in the tooling area.
7. Once the sheet starts sagging, activate the vacuum and promptly bring the PC sheet into contact with the mold for optimal forming.
8. Allow the sheet to cool before demolding. The mold can be reused in subsequent cycles.
9. Use heavy-duty scissors to trim excess edges from the formed part.
10. If the bottom of the PC part becomes foggy after several cycles, either switch molds or allow cooling between cycles, as this is typically due to uneven mold cooling and heat buildup over time.
11. Continue using the mold until signs of severe pitting or thermal cracking appear (molds generally last for at least 1,000 cycles)
